# Examining clinical correlates, treatment outcomes and mediators in young people with comorbid obsessive–compulsive disorder and autism spectrum disorder

**DOI:** 10.1007/s00787-021-01921-4

**Published:** 2021-12-16

**Authors:** A. D. Jassi, P. Vidal-Ribas, G. Krebs, D. Mataix-Cols, B. Monzani

**Affiliations:** 1 National and Specialist OCD, BDD and Related Disorders Clinic for Young People, South London and Maudsley NHS Foundation Trust, London, SE5 8AZ UK; 2 Social and Behavioral Science Branch, National Institute of Child Health and Human Development, National Institutes of Health, Bethesda, MD USA; 3 Social, Genetic and Developmental Psychiatry Centre, King’s College London, Institute of Psychiatry, Psychology and Neuroscience, London, SE5 8AF UK; 4 Centre for Psychiatry Research, Department of Clinical Neuroscience, Karolinska Institutet, Stockholm, Sweden

**Keywords:** Obsessive compulsive disorder, Autism spectrum disorder, Cognitive behaviour therapy, Insight, Family accommodation

## Abstract

Despite the high comorbidity, surprisingly little is known about the clinical features, treatment prognosis, and treatment mediators for youth with Obsessive–Compulsive Disorder (OCD) and Autism Spectrum Disorder (ASD). This study, the largest to date, compared 172 young people with OCD and ASD (OCD + ASD) to 447 without ASD (OCD) on clinical characteristics, finding those with OCD + ASD were more likely to endorse poorer insight into their OCD, have greater global functional impairment, greater levels of concurrent psychopathology, higher levels of family accommodation and to be on medication. Treatment outcomes following a course of Cognitive Behaviour Therapy with or without medication were explored for a subgroup; 100 young people with OCD + ASD and 223 with OCD. Whilst both groups benefitted from treatment, the OCD + ASD group had significantly poorer treatment outcomes. Greater global functional impairment and being on medication mediated the between-group difference in outcomes. Further research and treatment refinements are needed to improve outcomes for youth with OCD + ASD.

## Introduction

Obsessive–compulsive disorder (OCD) is a common psychiatric condition affecting approximately 0.25–3% of children and adolescents [1, 2]. It is marked by substantial distress and functional impairment, including poor educational, social, and/or family functioning [3, 4]. The prevalence of OCD is considerably higher among young people with autism spectrum disorders (ASD) than among the general population. A meta-analysis of data from 31 studies estimated the prevalence of OCD among youth with ASD to be over 17% [5]. Despite the high rates of comorbidity, little is known about the clinical presentation and treatment response of young people with OCD and ASD (hereafter referred to as ‘OCD + ASD’), compared to those without ASD (hereafter referred to as ‘OCD’).

Only a small number of studies have examined the clinical characteristics of youth with OCD + ASD, and findings have been inconsistent. No differences in OCD symptom severity [6–8] or insight [8] have been reported in studies comparing youth with OCD + ASD versus OCD. However, higher levels of functional impairment [8, 9] and family accommodation (FA) [8] in youth with OCD + ASD have been observed in some studies. High levels of FA have been consistently reported across the OCD literature [10], yet very few studies have examined FA in youth with ASD [8, 11]. FA has been found to predict treatment outcomes in young people with OCD [12–14] and those with OCD + ASD [15]. Additionally, youth with OCD + ASD have been found to have higher levels of comorbidity compared to those with OCD [8], specifically higher rates of diagnosed attention deficit hyperactivity disorder or inattention/hyperactivity symptoms [6–8], externalising disorders [8], peer problems [6] and social and separation anxiety [7]. Those with OCD + ASD have also been found to have lower prosocial behaviors compared to those with OCD [6]. Finally, some studies have found that individuals with OCD + ASD less frequently report sexual obsessions and checking, washing, repeating, games and superstitious compulsions [7, 8], whereas one study found no significant differences in OCD obsessions and compulsions [6]. Of note, most of the studies exploring clinical characteristics had relatively small sample sizes of youth with OCD + ASD (*n* = 12–70). Whilst Martin and colleagues [9] included a large cohort of young people (*n* = 7922), which is a strength of this study, our understanding of the wider clinical presentation of youths with OCD + ASD is nevertheless still limited as they used only a single item measure of global functioning. Given the limited available evidence, more research is needed to define and establish the OCD clinical phenotype of this common subgroup of patients. Clinical characterization of this group is important in order to address their needs, including tailoring therapeutic options.

Cognitive behavioral therapy (CBT) is the evidence-based psychological treatment for OCD [16–18]. Whilst single case studies/series and small trials (*n* = 1–58) have found adults and young people with OCD + ASD to benefit from CBT for OCD [19], there is indication in studies with small samples (*n* = 22–25) that the outcomes (OCD symptom reduction) may be poorer compared to typically developing youths with OCD [8, 20]. Yet, the reasons for the difference in outcomes, above and beyond having a diagnosis of ASD, remain unclear. Establishing the factors that account for the poorer OCD treatment outcomes in young people with OCD + ASD can provide a useful focus for clinical adaptations of current evidence-based practices to enhance treatment efficacy and ultimately patient outcomes.

Accordingly, the current study employed the largest sample to date of well-characterised youth with OCD and OCD + ASD to examine and compare these groups on clinical characteristics and treatment outcomes. The study also sought to investigate for the first-time putative mediators accounting for differences in CBT treatment outcomes between the two groups. Based on previous research, we expected higher levels of functional impairment [8, 9], family accommodation [8], and concurrent psychopathology [6–8] in the OCD + ASD group compared to the OCD group. We also predicted differences in OCD symptom dimensions between the groups, possibly the OCD + ASD group reporting less sexual obsessions and checking, washing, repeating, games and superstitious compulsions [7, 8]. Second, given the existing evidence thus far, it was hypothesized that youth with OCD + ASD would have poorer treatment outcomes than those with OCD at end of treatment [8, 20]. Finally, given the lack of available studies, no specific hypotheses were advanced with respect to variables that may mediate the differential response to treatment in these groups.

## Methods

### Participants

Participants were 619 young people aged 6–18 years (Mean = 14.6, SD = 2.20) referred to the National and Specialist OCD, BDD and Related Disorders Clinic for Young People at the Maudsley Hospital (London, UK) between 2005 and 2018. All participants met ICD-11 diagnostic criteria for OCD and completed the Children's Yale-Brown Obsessive–Compulsive Scale (CY-BOCS) at baseline [21]. The OCD diagnosis was confirmed by a specialist multidisciplinary team during a comprehensive assessment, as previously described [22]. Of the total sample, 172 participants (28%) also met ASD criteria. ASD diagnoses were typically assigned prior to the referral to the specialist OCD Clinic by a Consultant Psychiatrist in a community Child and Mental Health Service (CAMHS). In 68.6% (*n* = 118) of these cases, the ASD diagnosis was verified by a trained clinician completing a structured diagnostic instrument, namely the Autism Diagnostic Observation Schedule (ADOS) [23], Autism Diagnostic Interview-Revised (ADI-R) [24], or both. The remaining young people had their diagnosis given via clinician assessment without these structured measures. No participants had a diagnosed global learning disability.

A total of 323 (52.2%) young people received CBT at the specialist clinic and had post-treatment data available; the remaining (*n* = 296, 47.8%) received treatment elsewhere or post-treatment data was not available (e.g. treatment ongoing). CBT for OCD included three phases of treatment: psychoeducation on OCD and anxiety, exposure and response prevention (ERP) and relapse prevention [25]. Families were actively involved in sessions to address family accommodation and to support their child with in-session and homework ERP tasks. Information about medication was available for 296 (92%) of young people and missing for 27 (8%). Of those with medication information available, 168 (57%) received both CBT and medication. Of those on medication, information on type of medication was available for 110 (65% of those on medication); 109 participants were on Selective Serotonin Reuptake Inhibitors (SSRI) and 1 was on a medication other than SSRI. For the remaining 58 participants on medication (35%), information on their type of medication was not available.

All data used in the current study were collected as part of clinical practice. Young people and parents completed measures at baseline (first point of contact with clinic) and at the end of treatment (8–12 months after the baseline assessment). Data were then retrieved from medical records for the purposes of this study. Study approval was granted by the South London and Maudsley Child and Adolescent Mental Health Service Audit Committee.

### Measures

#### The Children's Yale-Brown Obsessive–Compulsive Scale (CY-BOCS)

The CY-BOCS is a widely used clinician-administered measure of OCD symptoms and severity [21]. It includes a checklist and 10 items assessing the severity of obsessions and compulsions (time spent, interference, distress, resistance and control), with total scores ranging from 0 to 40. The scale also measures insight into symptoms, using a 5-point scale ranging from 0 (excellent insight) to 4 (completely lacks insight). The CY-BOCS shows excellent psychometric properties with high inter-rater reliability and construct validity [21, 26]. In addition to the severity scores, the scale includes a symptom checklist to assess the presence or absence of common types of obsessions and compulsions, e.g. contamination obsessions, checking compulsions, etc. Symptom dimensions for this study were derived as described previous studies [6–8, 26, 27]. The rating for each dimension was dichotomous (i.e. yes or no) depending on whether at least one symptom was present within each dimension.

#### Children’s global assessment scale (CGAS)

This is an adaptation of the Global Assessment Scale for adults; it is a clinician-rated scale that measures global level of functioning across domains for children [28]. The scale ranges from 0 to 100 with higher scores indicating better functioning. The CGAS has shown good psychometric properties including good inter-rater reliability [28, 29].

#### Family accommodation scale-parent report version (FAS-PR)

This is a 13-item parent report questionnaire that measures the degree to which family members accommodate their child’s OCD symptoms and the level of distress or impairment that they experience as a result [30]. Each item is rated on a 5-point Likert scale ranging from 0 (never) to 4 (daily). The scale includes two subscales: involvement in compulsions and avoidance of triggers, as well as a total score [31]. Scores above 13 indicate clinically significant levels of family accommodation. The FAS-PR has demonstrated a stable factor structure, excellent internal consistency, good convergent validity, and adequate discriminant validity [31]. Given mothers completed this most consistently, only their measures were included in the current study.

#### Strengths and difficulties questionnaire (SDQ)

The SDQ is a 25-item questionnaire assessing child mental health difficulties incorporating five subscales capturing emotional difficulties, conduct problems, hyperactivity problems, peer problems, and pro-social behavior; a total difficulties score ranging from 0 to 40 is generated with the first four subscales. The measure is widely used across a range of clinical settings, and has been shown to have good psychometric properties, including good internal consistency and retest stability [32]. In the current study we used the parent reported SDQ to examine concurrent psychopathological symptoms.

### Statistical analyses

Data were analysed using Stata Version 16 (www.stata.com). Analyses were organized in three sections. First, we examined differences between young people with OCD and young people with OCD + ASD in key demographic and clinical variables employing independent *t*-tests and chi-square tests for continuous and categorical data, respectively. For these comparisons, we used Benjamini–Hochberg False Discovery Rate (FDR) correction at *p* < 0.05 to address multiple testing, which yielded an uncorrected alpha threshold of < 0.02.

Second, we compared treatment response between young people with OCD and young people with OCD + ASD in those with baseline and post-treatment outcome data available (*n* = 323, 52%) (i.e., scores on the CY-BOCS). To do so, a repeated-measures analysis of variance (ANOVA) was conducted with group as between-subject factor and time as within-subject factor, with CY-BOCS scores as dependent variable. Differences in rates of treatment responders, defined as a decrease in CY-BOCS of ≥ 35%, and remitters, defined as a post-treatment CY-BOCS score of ≤ 12 [26] were examined with logistic regression. The definitions of treatment response and remission employed in this study are considered the best suited for clinical-setting-based research [33] and have been shown to provide a proxy indicator for changes in quality of life in addition to symptom reduction [34].

Finally, we employed mediation analyses to examine the extent to which clinical variables that differed between the two groups explained the differential response to CBT for OCD. Specifically, we examined the mediating effects of sex, level of insight, functional impairment, family accommodation and medication status in explaining differences in treatment outcomes between youth with OCD and OCD + ASD. For a mediation to occur, the indirect effects should be significant; moreover, the proportion of the group effect on percentage of change in CY-BOCS scores during treatment that is mediated was calculated as the ratio between indirect effects and total effects.

## Results

### Demographic and clinical characteristics of youth with OCD versus OCD + ASD

Table 1 provides descriptive statistics of demographic and clinical variables for each group, as well as comparison analyses statistics. Groups did not statistically differ in age, age at onset of OCD, family history of OCD, CY-BOCS scores (total, obsessions and compulsions) and conduct problems. However, compared with youth in the OCD group, those in the OCD + ASD group were more likely to be males (OR = 0.52, 95% CI 0.36, 0.75), to present with poorer insight (*d* =  – 0.30, 95% CI  – 0.53–0.07), greater functional impairment (*d* = 0.40, 95% CI 0.21–0.59), family accommodation (*d* =  – 0.47, 95% CI  – 0.69 to 0.26), and to be on medication (OR = 2.78, 95% CI 1.81, 4.27). In addition, youth with OCD + ASD had lower rates of aggressive (OR = 0.45, 95% CI 0.27, 0.75) and religious obsessions (OR = 0.47, 95% CI 0.28, 0.79) and game/superstitious compulsions (OR = 0.54, 95% CI 0.27, 0.90), than youth with OCD. Youth with OCD + ASD also presented with more psychopathological symptoms as measured by the SDQ (*d* =  – 0.74, 95% CI  – 0.98,  – 0.50), including emotional (*d* =  – 0.28, 95% CI  – 0.52,  – 0.06), hyperactivity (*d* =  – 0.40, 95% CI  – 0.61,  – 0.19) and peer problems (*d* =  – 0.79, 95% CI  – 1.00,  – 0.58), and less prosocial behaviours (*d* = 0.63, 95% CI 0.40, 0.87).

**Table 1:** Clinical characteristics in OCD participants with and without ASD

	OCD *N *= 447	OCD+ASD *N* = 172	*t*	*p*-value	
	*M (SD) n*	*M (SD) n*			*ES (Cohen’s d) 95%CI*
Age, years	14.6 (2.3) 436	14.6 (2.1) 170	0.19	0.852	0.02 ( – 0.16, 0.19)
Age of onset	11 (3.2) 311	10.5 (2.8) 80	1.25	0.212	0.16 ( – 0.09, 0.40)

	*n (%) den.*	*n (%) den.*	*χ*^*2*^	*p*-value	*ES (Odd Ratio) 95%CI*
Gender, females	229 (51.2) 447	61 (35.5) 172	12.40	**< 0.001**	**0.52 **(0.36, 0.75)
Medication	219 (54.5) 402	113 (76.9) 147	22.58	**< 0.001**	**2.78 **(1.81, 4.27)
Family hist. OCD	64 (23.1) 277	13 (23.2) 56	0.00	0.986	1.01 (0.51, 1.99)

	*M (SD) n*	*M (SD) n*	*t*	*p*-value	*ES (Cohen’s d) 95%CI*
FAS-PR					
Total	25.5 (13) 299	31.5 (11.2) 115	– 4.32	**< 0.001**	– **0.47** ( – 0.69, – 0.26)
Avoidance	11.5 (7.0) 305	14.4 (6.8) 106	– 3.60	**< 0.001**	– **0.41** ( – 0.63, – 0.18)
Involvement	14.1 (6.8) 300	16.8 (5.8) 105	– 3.69	**< 0.001**	– **0.42** ( – 0.64, – 0.19)
CGAS	43.7 (9.6) 358	40.0 (5.6) 147	4.10	**< 0.001**	**0.40** (0.21, 0.59)
SDQ					
Total difficulties	16.7 (6.0) 264	21.2 (6.4) 98	– 6.27	**< 0.001**	– **0.74 (** – **0.98, ** – **0.50)**
Emotional problems	6.3 (2.6) 269	7.0 (2.5) 99	– 2.44	**0.015**	– **0.28 (** – **0.52, ** – **0.06)**
Hyperactivity problems	5.0 (2.6) 320	6.0 (2.7) 123	– 3.79	**< 0.001**	– **0.40 (** – **0.61, ** – **0.19)**
Conduct problems	2.3 (1.8) 323	2.5 (1.7) 124	– 1.13	0.257	– 0.12 ( – 0.33, 0.09)
Peer problems	3.13 (2.4) 319	5.0 (2.4) 124	– 7.48	**< 0.001**	– **0.79 (** – **1.00, ** – **0.58)**
Prosocial behaviors	7.2 (2.2) 268	5.7 (2.6) 97	5.36	**< 0.001**	**0.63 (0.40, 0.87)**
CY-BOCS					
Total	27.6 (4.9) 447	28.0 (4.9) 172	– 1.08	0.282	– 0.10 ( – 0.27, 0.08)
Obsessions	13.5 (2.6) 447	13.8 (2.8) 171	– 1.32	0.188	– 0.12 ( – 0.29, 0.06)
Compulsions	14.1 (2.7) 447	14.2 (2.4) 171	– 0.65	0.518	– 0.06 ( – 0.23, 0.12)
Insight	1.7 (1.1) 233	2.0 (1.0) 105	– 2.56	**0.011**	– **0.30 (** – **0.53, ** – **0.07)**

	*n (%) den.*	*n (%) den.*	*χ*^*2*^	*p*-value	*ES (Odd Ratio) 95%CI*
CY-BOCS Obsessions					
Contamination	246 (73) 337	73 (82) 89	3.05	0.081	1.68 (0.93, 3.05)
Aggressive	269 (79.8) 337	57 (64) 89	9.76	**0.002**	**0.45 (0.27, 0.75)**
Sexual	100 (29.4) 340	29 (31.9) 91	0.21	0.649	1.12 (0.68, 1.85)
Hoarding	104 (30.9) 337	23 (25.3) 91	1.07	0.301	0.76 (0.45, 1.28)
Magical thoughts	133 (39.5) 337	28 (30.8) 91	2.31	0.129	0.68 (0.42, 1.12)
Somatic	127 (37.7) 337	27 (30) 90	1.82	0.177	0.71 (0.43, 1.17)
Religious	145 (43) 337	24 (26.4) 91	8.32	**0.004**	**0.47 (0.28, 0.79)**
CY-BOCS Compulsions					
Cleaning	258 (76.8) 336	78 (85.7) 91	3.40	0.065	1.81 (0.96, 3.44)
Checking	264 (78.8) 335	68 (74.7) 91	0.69	0.405	0.80 (0.46, 1.37)
Repeating	221 (66.2) 334	58 (63.0) 92	0.31	0.577	0.87 (0.54, 1.41)
Counting	165 (49.0) 337	38 (41.3) 92	1.70	0.192	0.73 (0.46, 1.17)
Ordering/Arranging	168 (50.2) 335	92 (52.2) 92	0.12	0.731	1.08 (0.68, 1.72)
Hoarding	117 (34.7) 337	31 (33.7) 92	0.03	0.855	0.96 (0.59, 1.56)
Games/Superstitious	138 (41.1) 336	25 (27.5) 91	5.61	**0.018**	**0.54 (0.27, 0.90)**

*ASD* autism spectrum disorder, *CGAS* Children’s Global Assessment Scale; *CY-BOCS* Children's Yale–Brown Obsessive–Compulsive Scale, *den*. denominator, *ES* effect size, *FAS-PR* Family Accommodation Scale-Parent Report, *SDQ* Strengths and Difficulties Questionnaire, *hist*. history, *M* mean, *OCD* obsessive compulsive disorder, *SD* standard deviation

Bold *p*-values and effect sizes indicate significance after False Discovery Rate (FDR) correction for multiple testing

### Treatment outcomes for youth with OCD versus OCD + ASD

There was no statistical difference in the proportion of participants with treatment outcome data available across groups (OCD, *n* = 223 [50%] vs OCD + ASD, *n* = 100 [58%]; χ^2^(1) = 3.39, *p* = 0.066). Those with and without available treatment data did not statistically differ with respect to sex, mean age, age of onset of OCD, rates of family history of OCD, rates of prescribed medication, CY-BOCS scores, SDQ total difficulties, and SDQ scores in the subscales of emotional problems, hyperactivity problems, peer problems and prosocial behaviours. However, those with available treatment outcome data had lower scores on family accommodation as measured with FAS-PR (t(412) = 2.52, *p* = 0.012), lower scores on conduct problems as measured with the SDQ (t(445) = 2.36, *p* = 0.019) and higher scores on measures of functional impairment (i.e. CGAS) (t(503) =   – 3.69, *p* < 0.001) than those with no treatment data. Both groups attended up to 20 CBT sessions, with the OCD group attending a mean of 13.69 (SD = 5.56) and OCD + ASD group attending a mean of 15.74 (SD = 5.61). There were no significant differences between groups in the number of CBT sessions attended (t(321) =   – 2.15, *p* = 0.380).

**Fig. 1:**
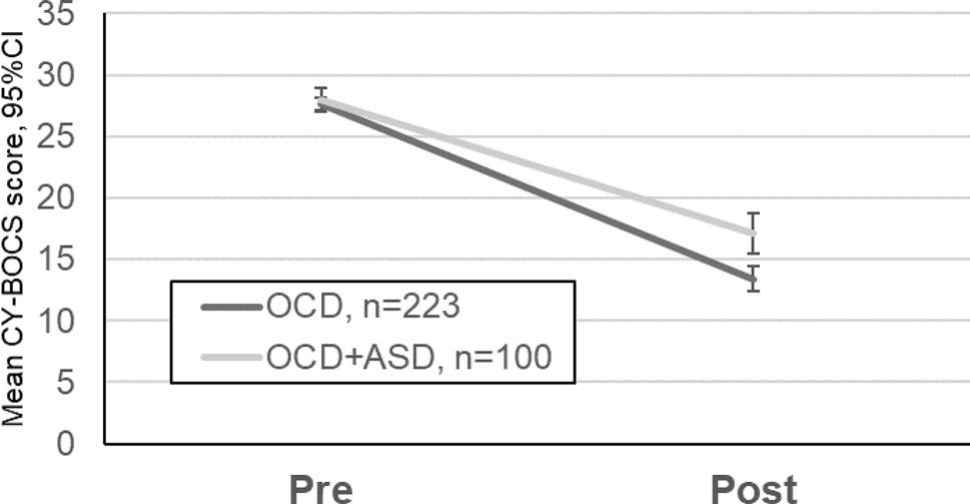
Mean CY-BOCS score and their 95% confidence intervals by group before and after treatment. *CI* confidence interval, *CY-BOCS* Children's Yale-Brown Obsessive-Compulsive Scale, *OCD* obsessive compulsive disorder

As expected, results for repeated measures ANOVA revealed that both groups improved significantly (OCD: F(1,642) = 539.37, *p* < 0.001; OCD + ASD: F(1,642) = 143.98, *p* < 0.001); however, there was a group by time interaction (F(1,642) = 8.72, *p* = 0.003) in which those youth with OCD + ASD showed less improvement than those with only OCD at post-treatment (F(1,642) = 22.34, *p* < 0.001) (Fig. 1).

In terms of treatment responders (i.e., ≥ 35% reduction on CY-BOCS) and remitters (i.e., CY-BOCS score ≤ 12 the end of treatment), 53% of participants from the OCD + ASD group responded to treatment compared to 76.7% of the OCD group (OR = 0.34, 95% CI 0.21, 0.57, *p* < 0.001); with similar rates for males (53.2% vs 80.2%, OR = 0.28, 95% CI 0.14, 0.56, *p* < 0.001) and females (52.6% vs 73.2%, OR = 0.41, 95% CI 0.19, 0.87, *p* = 0.021). In terms of remission 31% of participants remitted in the OCD + ASD group compared to 49.8% in the OCD group (OR = 0.45, 95% CI 0.28, 0.75, *p* = 0.002 with similar rates for males (30.7% vs 53.2%, OR = 0.39, 95% CI 0.20, 0.75, *p* = 0.005) and females (31.6% vs 46.4%, OR = 0.53, 95% CI 0.24, 1.16, *p* = 0.113).

### Putative mediators explaining differences in treatment outcomes

Table 2 provides the results for the mediation analyses, in which we examined the mediating roles of sex, levels of insight, family accommodation, functional impairment, medication status and overall concurrent psychopathological symptoms. These factors were selected for analysis in light of the significant differences observed between the two groups in this study. The mediation effects of functional impairment and medication status were statistically significant; specifically, the proportion of the group effect on differences in treatment response that was mediated by functional impairment and medication status was 19% and 31%, respectively. In other words, the lower treatment response observed in youth with OCD + ASD was partially explained by their higher functional impairment and being on prescribed medication. In contrast, sex, family accommodation, levels of insight and concurrent psychopathological symptoms did not mediate group differences in treatment outcomes (Table 2).

**Table 2:** Direct, indirect (mediated), and total effects of group on percentage of change of CY-BOCS scores between pre- and posttreatment

Mediator	*n*	Direct effects			Indirect effects			Total effects		
		b	SE	*p*-value	b	SE	*p*-value	b	SE	*p*-value
Sex	323	– 0.12	0.03	** < 0.001**	0.00	0.00	0.682	– 0.12	0.03	** < 0.001**
Insight	187	– 0.07	0.04	0.085	– 0.01	0.01	0.150	– 0.09	0.04	**0.042**
FAS-PR	236	– 0.11	0.04	**0.004**	– 0.02	0.01	0.128	– 0.13	0.04	**0.001**
CGAS	299	– 0.10	0.03	**0.003**	– 0.02	0.01	**0.020**	– 0.12	0.03	** < 0.001**
Medication	296	– 0.07	0.04	**0.049**	– 0.03	0.01	**0.010**	– 0.10	0.04	**0.004**
SDQ Total	202	– 0.08	0.04	0.060	– 0.00	0.02	0.948	– 0.08	0.04	0.040

*B* unstandardized coefficient, *CGAS* Children’s Global Assessment Scale, *CY-BOCS* Children's Yale-Brown Obsessive–Compulsive Scale, *FAS-PR* Family Accommodation Scale-Parent Report, *SDQ* Strengths and Difficulties Questionnaire, *SE* standard error. Direct effects represent the effects of group adjusted for the mediator. Indirect effects represent the mediated effects of group. Total effects represent the sum of direct and indirect effects. Bold *p*-values indicate significance

## Discussion

To our knowledge, this is the largest study to date comparing the clinical phenotype and treatment outcomes for young people with OCD with and without comorbid ASD using a broad range of outcome measures and the first one to examine factors that may explain differences in treatment outcomes, above and beyond having a diagnosis of ASD.

Regarding the clinical presentation, both groups showed similar levels of overall OCD severity, including severity of obsessions and compulsions. Together with previous studies [6–8], such findings suggest that OCD affects individuals with ASD to a similar degree to youth without ASD, with both groups endorsing equally time-consuming, distressing and interfering obsessions and compulsions. However, a number of differences in clinical characteristics were observed in this study. Specifically, those with OCD + ASD presented with higher levels of family accommodation, poorer insight into their OCD, worse global functioning, higher levels of comorbid psychopathology, in particular hyperactivity, emotional and peer problems, lower levels of prosocial behavior and were more likely to be prescribed medication than those with OCD without ASD.

Results regarding family accommodation are consistent with the high rates of parental accommodation of OCD symptoms as well as parental accommodation of restrictive and repetitive behaviors (RRBs) reported previously in samples of youth with ASD and those with comorbid OCD and ASD [8, 11]. The finding that people with ASD + OCD had higher levels of family accommodation might be due to parents accommodating both RRBs and OCD symptoms. Alternatively, given parents may be accustomed to accommodating their child's ASD symptoms, it makes it easier for them to accommodate OCD. Equally, parents’ knowledge and skills to distinguish RRBs from OCD compulsions may contribute to higher parental accommodation. Family accommodation may be an important avenue to address in future research and in clinical settings; for example, a better understanding of the factors that drive or maintain family accommodation in parents of children with OCD + ASD may help address the needs and difficulties of youth with this comorbidity. Albeit preliminary, the findings highlight avenues for potential additional parent-based intervention and guidance for how to address RRBs versus OCD rituals.

It is noteworthy that youth with OCD + ASD presented with poorer insight into their OCD symptoms compared to youth without ASD. This novel finding contrasts with that of Griffiths and colleagues [8] who reported no significant difference in insight despite using the same measure. Due to the smaller sample (*n* = 25 in each group) their study may have been underpowered to detect small effect sizes as were observed for insight in the current study. Whilst in need of replication, this finding might be associated with cognitive rigidity and difficulties with metacognition often reported in ASD, leading to difficulties in acknowledging the excessive and unreasonable nature of their obsessions and compulsions. Further research is needed to explore insight among those presenting with OCD and ASD. Consistent with previous studies [8, 9], the OCD + ASD group had greater functional impairment compared to the OCD group. However, what remains to be examined is how much of the impairment is due to OCD versus ASD as the measure used in this study only captured overall functional impairment. It is likely that there is a cumulative burden of having co-occurring OCD and ASD, which may be further compounded by the elevated levels of other psychopathological difficulties (e.g. attentional problems) that were found in the current study. It would be important for future research to investigate functional impairment among youth with co-morbid OCD and ASD in greater depth, for example, using assessment tools that allow to delineate OCD- versus ASD-related impairment. Whilst Griffiths and colleagues [8] measured OCD-related functional impairment, they did not examine ASD-related impairment. Knowing how much each of these diagnoses contribute to greater functional impairment may give ideas of avenues of support for those with a dual diagnosis. Additionally, it would be interesting to explore the impact of specific autistic symptoms on OCD treatment outcomes and functional impairment, including the severity of impairments in social communication, and repetitive and restricted behaviors.

Like previous studies, we found higher rates of hyperactivity symptoms [6–8], peer problems [6], emotional problems [7] and lower prosocial behaviors [6] in young people with OCD + ASD compared to those with OCD. Contrary to previous research [8], we failed to find differences in conduct problems. The latter may be characteristic of the sample seen in the clinic and would need to be replicated to ascertain whether this is a clinical characteristic of those with OCD + ASD. Overall, these results are unsurprising as we know young people with ASD are likely to have high levels of psychopathology [35]. The finding that youth of OCD + ASD had higher peer problems and lower prosocial behaviour is characteristic of the ASD profile [36]. These findings have important clinical implications in that they highlight that youth with OCD + ASD will present with additional difficulties; however, the finding that this does not impact on CBT outcomes (discussed below) is important as it highlights that this may not need to be specifically addressed to treat OCD symptoms.

Finally, the reason for increased use of medication in those with a dual diagnosis is unclear since both groups showed a comparable severity of OCD symptoms. It is possible that those with OCD + ASD are more likely to be prescribed medication due to the greater functional impairment or due to a poor or partial response to previous courses of CBT. Alternatively, it is possible that clinicians may be more inclined to offer medication to those presenting with this dual diagnosis [9]. Further research is warranted to clarify the treatment history of those with comorbid OCD + ASD which may help understand reasons for increased medication use and its potential impact on CBT outcomes.

Consistent with a previous investigation [8], we found young people with OCD + ASD reported fewer games/superstitious compulsions compared to the OCD group. This may be due to young people with ASD struggling with abstract thinking such as that involved in superstitious related compulsions. The current study also found the OCD + ASD group reported fewer aggressive and religious obsessions, which has not been reported before. This is at odds with studies that have found no differences in symptom dimensions between the groups [6] or differences found in other dimensions, namely sexual obsessions, checking, washing and repeating compulsions [7]. It is difficult to ascertain what may account for differences across studies as they all used the same measure (CY-BOCS). Given the inconsistencies in the literature, our findings are in need of replication in future research.

Both groups showed a reduction in OCD symptoms after treatment. However, as hypothesized, and in line with previous research, the OCD + ASD group showed less improvement at the end of treatment compared to the OCD group [20]. This was echoed with lower rates of response and remission rates in the OCD + ASD group compared to the OCD group. The response rate in the OCD + ASD group found in this study (51%) was broadly comparable to those reported in the adult and pediatric literature, whereby rates range from 45% [15] to 66% [37]. The remission rates in the current study (31%) were also found to be in line with previous studies, ranging from 9% [20] to 52% [37]. Our findings provide additional evidence in a naturalistic setting in support of CBT for OCD as an effective treatment for youth people with OCD + ASD. In addition, this study supports previous research showing a reduced treatment response among youth with ASD + OCD and highlights the continued need for rigorous research to optimize CBT treatment for those with this common dual diagnosis [8, 9, 20]. Several approaches have been suggested to enhance outcomes in this challenging group, including specific ASD-adaptations, increasing the quantity and intensity of CBT sessions and delivering home-based sessions [11, 38–40].

Multiple mediators were examined in an attempt to explore which factors, over and above having a diagnosis of ASD, may account for the group difference in treatment outcomes. Global functional impairment and being on medication were found to partly account for the poorer treatment outcomes in youth with OCD + ASD compared to those with OCD only. Indeed, we found that functional impairment mediated 19% of the effect of a dual diagnosis on CBT outcomes; an even higher proportion of the difference in outcomes was mediated by being on medication (31%). This is a novel finding that encourages more clinical consideration and research. It is possible that those with co-occurring OCD + ASD may have a cumulative burden of having both OCD and ASD, causing greater functional impairment and resulting in poorer CBT outcomes. This raises the question of whether promoting functioning (e.g. optimizing educational support, facilitating engagement in leisure/social activities) could provide a helpful platform for CBT to enhance outcomes. Being on medication also mediated the differential treatment outcomes. It is possible medication may be a proxy for a measure of a complexity (e.g. poorer insight, treatment resistance) that was not captured in this study and that interferes with treatment outcome. Taken together, our results provide preliminary support in favor of further examination of functional impairment and pharmacological approaches for young people with OCD + ASD.

Surprisingly, family accommodation did not account for differential treatment outcomes between those with OCD versus OCD + ASD, despite the significant group differences in family accommodation and previous studies showing family accommodation to predict CBT outcomes [12–15]. The current findings may reflect that family accommodation was successfully addressed in treatment; a pre- and post-treatment measure of family accommodation would have been helpful to ascertain this and could be considered in future research. Insight was significantly poorer in the OCD + ASD group and has in previous studies been found to predict treatment outcomes [41]. However, the fact that insight did not account for differential treatment outcomes may be due to insight being measured on a single-item scale of 0–4, so there potentially was not sufficient variance to fully test this association. Future research should utilize more detailed measures of insight, such as the Brown Belief Assessment Scale (BABS) [42]. Alternatively, insight could have improved over the course of treatment as has been found in previous studies [41]. Limited by sample size, our analyses were unpowered to test treatment group by sex interactions in response to treatment. Future studies should properly examine the role of sex in this effect. It is interesting to find that concurrent psychopathology did not predict CBT outcomes. This  highlights that having ASD may  account for compromised outcomes as opposed to comorbidities, indicating that treatment needs to be modified to support this group with the aspects of treatment they may struggle with, e.g. extended psychoeducation on emotions for those with alexithymia.

The present findings have important implications. Clarifying the association between ASD, functional impairment and medication prescription could be an important direction for future research. The finding that functional impairment and being on medication emerged as mediators for the difference in CBT outcomes for youth with OCD + ASD also suggests that these may be important avenues for improving outcomes for this patient group. A better understanding of such constructs will help refine our OCD treatment protocols for this patient group, for instance, introducing elements to better address impairments associated to ASD (e.g. sensory sensitivity, emotion psychoeducation) or dropping other elements that do not contribute to change. Indeed, the treatment focus is typically on reducing OCD symptom severity; however, the results may highlight the potential value of improving functioning to enhance treatment outcome.

There are several limitations to the current study. The study was conducted in a National and Specialist clinic, so there may be a bias in referrals where the OCD is the most prominent issue and, therefore, we may not have seen a full range of young people on the autism spectrum; most notably, we did not have young people with global learning disabilities. We may have also had a higher proportion of young people with more severe and treatment resistant OCD referred to us compared to other studies, which may have impacted our results. Only a subgroup of the sample had treatment outcome data available. Although those with and without treatment data did not differ on many clinical characteristics (e.g. age of onset,  CY-BOCS scores etc),  those with data had lower levels of family accommodation, conduct problems and functional impairment, representing possibly a less severe subgroup of patients. The lack of information on medication for 8% of young people and the type of medication (SSRIs or other) for 35% also limits our conclusions regarding the role of medication in mediating outcomes. Another limitation is the lack of a control group (e.g. OCD with another comorbidity) which did not allow us to explore if the results were specific to having an ASD comorbidity versus having any comorbidity. This would be important to include in future research. The lack of structured diagnostic assessments of comorbidities in this study is a further limitation. Additionally, the measure of family accommodation measure was self-report and, therefore, it may be difficult to establish if it captured family accommodation of OCD symptoms only or whether parents included ASD symptoms too. The measure of functional impairment was a clinician-rated global measure, which did not allow us to distinguish disorder-specific impairment nor to get parent or child reports of impairment. Finally, OCD assessments were carried out by expert clinicians specialised in OCD and experienced in assessing and disentangling ASD ego-syntonic RRBs from ego-dystonic OCD compulsions; however, we cannot completely rule out that CY-BOCS may have been influenced by RRBs. A measure such as the ASD modified version of the CY-BOCS [43] could be used in future studies to help address this potential limitation.

Despite these limitations, the present study remains the largest study to date comparing the clinical characteristics and treatment outcomes for youth with OCD + ASD compared to those without ASD. To the best of our knowledge, this is also the first study to examine putative mediators accounting for the difference in treatment outcomes between youth with OCD + ASD versus OCD only. The results highlight that the presentation of OCD in those with comorbid OCD and ASD does not significantly differ from those without ASD in terms of symptom severity. However, those with comorbid OCD + ASD present with higher levels of family accommodation, functional impairment and poorer insight and are more likely to be on medication. Those with OCD + ASD also present poorer treatment outcomes compared to youth with OCD, with functional impairment and medication status emerging as significant mediators of outcome differences and as potential avenues in future research on the development and adaptation of treatment for those with comorbid OCD and ASD.

## Declarations

### Availability of data and material

N/A.

### Code availability

N/A.
